# Value creation of copra meal mannan into functional manno-oligosaccharides (β-MOS) using the mannanase *Bacillus* man B (*Bl*Man26B)

**DOI:** 10.1038/s41598-024-73255-5

**Published:** 2024-09-27

**Authors:** Nguyen Cao Cuong, Dietmar Haltrich, Thae Thae Min, Thu-Ha Nguyen, Montarop Yamabhai

**Affiliations:** 1 Faculty of Engineering and Food Technology, Hue University of Agriculture and Forestry, Hue University, Hue, 530000 Thua Thien Hue Vietnam; 2 Molecular Biotechnology Laboratory, School of Biotechnology, Institute of Agricultural Technology, Suranaree University of Technology, Nakhon Ratchasima, 30000 Thailand; 3 Food Biotechnology Laboratory, Department of Food Science and Technology, BOKU University, Vienna, 1190 Austria

**Keywords:** Defatted-copra meal, *Bacillus licheniformis* β-mannanase, Manno-oligosaccharides, β-MOS, Functional, Biotechnology, Molecular biology

## Abstract

Agricultural wastes rich in β-mannan are an important environmental problem in tropical and sub-tropical countries. This research aims at dealing with this and investigates the valorization of mannan-rich copra meal from virgin coconut oil manufacturing into mannan-oligosaccharides (β-MOS) by enzymatic hydrolysis using β-mannanase from *Bacillus licheniformis* (*Bl*Man26B). Lab-scale process, involving pre-treatment and bioconversion steps, were conducted and evaluated. Lyophilized β-MOS was analyzed and its biological activities were assessed. The size of oligosaccharides obtained ranged from dimers to hexamers with 36.7% conversion yields. The prebiotic effects of β-MOS were demonstrated in comparison with commercial inulin and fructo-oligosaccharides (FOS). In vitro toxicity assays of β -MOS on human dermal fibroblasts and monocytes showed no cytotoxic effect. Interestingly, β-MOS at concentrations ranging from 10 to 200 µg/mL also demonstrated potent anti-inflammatory activity against LPS-induced inflammation of human macrophage THP-1 in a dose-dependent manner. However, at high dose, β-MOS could also stimulate inflammation. Therefore, further investigation must be conducted to ensure its efficacy and safe use in the future. These results indicate that β-MOS have the potential to be used as valued-added health-promoting nutraceutical or feed additive after additional in-depth studies. These finding should be applicable for other agricultural wastes rich in mannan as well.

## Introduction

Agricultural wastes rich in polysaccharides are promising sources of raw materials for bioconversion into high-value oligosaccharides with different functional activities, depending on the nature of the biopolymers from various sources. Adding value to agricultural waste has become an important issue for sustainable economy and the environment around the world^1^. The demand for nutraceutical and functional foods poses challenge and opportunity for value creation of various agricultural wastes. Particularly, high molecular weight (HMW) carbohydrates from various waste biomasses, such as cellulose, inulin, pectin, chitin/chitosan, hyaluronic acid, have been recycled for high-value innovative applications in pharmaceuticals, nutraceuticals, cosmetics and functional foods industries^2^. Enzymes as biocatalysts are the key elements of green and clean technology to create competitive innovations that are environmentally friendly, highly suitable for sustainable bio, circular, and green economy^3^.

Coconut tree (*Cocos nucifera* L.), belonging to the palm tree family, is prevalent in tropical and subtropical regions. It is utilized for various purposes, ranging from food to cosmetics. The inner flesh of the mature coconut, called copra, is used to extract coconut oil and milk. Coconut oil and coconut milk are commonly used in food preparation, soap making, as well as cosmetics^4^. It is the sixth most cultivated fruit in the world, producing approx. 63 million tons of nuts with a market value of USD 9.7 billions and its trade is mainly in the form of desiccated coconuts and coconut oil^5^. In a dried copra meal, about 46.62 ± 1.03% are carbohydrates, 22.94 ± 1.34% is crude protein, 13.04 ± 3.59% is crude fiber and 9.00 ± 4.05% is crude fat, of which the non-starch polysaccharide (mannan) is the predominant constituent^6^. Recently, the extraction of virgin coconut oil has gained more interest as it manifests several beneficial effects on consumers’ health^7^. From these processes, large amounts of cheap but high-quality organic residual materials are produced. The coconut cake or copra meal after oil and milk extractions is considered an agricultural by-product containing mainly of mannan (61%) and other polysaccharides such as cellulose, arabinoxylogalactan, and galactomannan^8^. Mannan is the second most abundant hemicellulose in nature, existing as structural and storage polysaccharides in higher plants including coconut. Linear mannan, or β-1,4 mannan, is a polymer of D-mannose units linked by β-1,4 glycosidic bond; while galactomannan comprises D-mannose moieties, linked by β-1,4 glycosidic bond as a backbone, and D-galactose, linked by β-1,6 glycosidic bond as branch chains, with more than 5% of galactose (w/w)^9^. Mannan is the major polysaccharide in copra meal, but the detailed characterization is not well known. The abundance of mannan makes copra meal an attractive raw material for bioconversion of a by-product into functional manno-oligosaccharides (MOS) by enzymatic hydrolysis. Manno-oligosaccharides seem to be particularly interesting as mannose is also one of the key sugars in the glycosylation process of human proteins that are related to the status of human health and disease^10^. β-Mannanase (EC 3.2.1.78) is an enzyme that randomly catalyzes the hydrolysis of β-1,4 glycosidic linkages in the main chains of β-1,4 mannans, glucomannans, and galactomannans. Mannanase can be produced by bacteria (*Bacillus* sp., *Lactobacillus* sp.), fungi (*Aspergillus* sp., *Trichoderma* sp.), or actinomycetes (*Streptomyces* sp.)^11^. Among them, fungi are known as the main sources of mannolytic enzymes. According to CAZy database, β-mannanase from *B. licheniformis* belongs to the glycoside hydrolase family GH26; hence, *Bl*Man26B. It possesses high stability at increased temperatures and in an alkaline condition, making it suitable for industrial use^12^. The enzyme can be efficiently produced and secreted from *Escherichia coli*^13^ and *Lactobacillus plantarum*, a food-grade expression host^14^. In this research, the conversion of copra meal from a coconut factory into value-added hydrolytic products using the enzyme *Bl*Man26B is demonstrated. Since the manno-oligosaccharides (MOS) were created from a mannan biopolymer comprising β-1,4-glycosidic backbone, the MOS products will be called β-MOS in this report to differentiate them from α-MOS obtained from yeast cell walls, which primarily comprise a polymer consisting of α-1,6-linked D-mannose^9^ or from mycelium cell wall (*Aspergillus fumigatus*), which consists of α-1,6-linked D-mannose and α-1,2-mannosyl residues in the backbone^15^. We hypothesized that in addition to its known prebiotic activity, MOS may have anti-inflammatory as previously shown for chito-oligosaccharides (CHOS) as well^16^.

This is the first report on the anti-inflammatory activity of β-MOS on human cell lines, in addition to its known prebiotic activity and safety. In this study, the whole process from the extraction of mannan from copra meal to bioconversion into value-added manno-oligosaccharides are described. The results should pave a way to more research and development on the bioconversion of mannan-rich agricultural wastes into valued-added health-promoting nutraceutical or feed additive in the future.

## Materials and methods

### Pre-treatment and composition of defatted copra meal

Copra meal obtained from a virgin coconut oil factory in Samut Sakhon province (see inlets of Fig. 5), Thailand was dried for 12 h at 60 °C and kept in a bag with vacuum seal for storage at 4 °C until further experiments as previously described^17^. The fat content was removed according to previously published protocol with minor modifications^18^. In brief, approximately 5 g of copra meal was boiled in 100 mL of DI water for 2 h and then cooled to 4–5 °C to solidify the oil. After the solidified oil was removed, this process was repeated two more times. Then, the sample was dried overnight at 60 °C in a hot air oven. Next, 1 L of *n*-hexane was used to suspend the copra meal under agitation overnight. Then, the solid part was collected by vacuum filtration, followed by overnight drying at 60 °C. The resulting product, termed defatted copra meal (DCM), was then crushed, sieved using a 0.2 mm mesh, and stored at 4 °C for the next experiments. Moisture and lipid content of DCM was determined using the AOAC methods^19^. The monosaccharide composition of DCM was measured by a two-step hydrolysis method^20^. The sample, after two hydrolysis steps with 77% and 25% sulfuric acid, was neutralized with an appropriate quantity of 0.05 M Ba(OH)_2_. The clear supernatant solution containing the monosaccharides was separated from the precipitated BaSO_4_ by decanting. The monosaccharides profile of DCM was analyzed with a HPLC system, consisting of an Aminex HPX42A column (Bio-rad) along with a refractive index detector from Hitachi Ltd., Japan. To ensure optimal separation, the column was set at 45 °C, using ultra-pure water as a mobile phase, at a flow rate of 0.6 mL/min. Glucose, xylose, mannose, and arabinose were used as standards in the HPLC analysis.

### Enzyme expression and purification

The expression of *Bl*Man26B was conducted according to our established procedure^12^. Briefly, single colonies of freshly transformed *E. coli* TOP10 were picked and cultured in 5mL of LB containing 100 µg/mL ampicillin at 37 °C, 250 rpm for 16 h. For 250-1000 mL of enzyme expression, 1 mL of culture was seeded into LB media and cultured to reach an OD_600_ of approximately 1.0–1.5. Then, 0.1 mM IPTG was added and further incubated at 26–28 °C, 250 rpm, for 3–4 h. Next, cells and supernatant were harvested by centrifugation at 2000 × *g* for 10 min at 4 °C. Both cell pellets and supernatant were kept at 4 °C for the next purification step using immobilized metal affinity chromatography (IMAC). The Ni-NTA agarose beads preincubated with periplasmic extract, prepared as previously published^21^, or culture supernatant were transferred onto a gravity-flow chromatography column and washed thrice using imidazole solutions (30 mM Tris–HCl, 300 mM NaCl, containing increasing amount of imidazole at 5 mM, 10 mM, and 20 mM) to remove non-specifically bound proteins. The purified β-mannanase was subsequently eluted with elution buffer (30 mM Tris–HCl, 300 mM NaCl, 250 mM imidazole) and dialyzed in a dialysis buffer (200mM NaCl in 20mM Tris-HCl, pH 8), using a 10-kDa MWCO snakeskin membrane.

### TLC analysis of hydrolytic products

To prepare the substrate used in the bioconversion reaction for TLC analysis, DCM corresponding to approximately 1%, 2%, and 4% (w/v) of mannan was resuspended in DI water. The MOS bioconversion reaction was conducted in 10 mL of various amount of substrate with varying concentrations of β-mannanase ranging from 5, 10, and 15 U/mL, at 40–50 °C, 250 rpm for 24 h. For TLC analysis, 850 µL of the reaction mixture was collected after 1, 2, 3, 6, 9, 12, 16, 20, and 24 h of incubation. The samples were boiled at 100 °C for 20 min, and then centrifuged. After that 0.25 µL of the samples, along with oligosaccharides standard M1 to M6 (Megazyme, Ireland), were spotted onto a TLC silica sheet (Silica gel 60 F254, Germany). The TLC sheets were developed using a mobile phase composed of ethanol, *n*-propanol, and water at a ratio of 4:14:2. To visualize the hydrolytic products, the TLC plates were sprayed with a developing solution (0.5w/v of thymol in a mixture of ethanol and H_2_SO_4_), followed by heating at 120 °C for 15–20 min.

In addition to TLC, the product was quantified using high-performance anion-exchange chromatography with pulsed amperometric detection (HPAEC-PAD), using a 4 × 250 mm DIONEX analytic column and ED40 electrochemical detection. To prepare the samples, 50 µL of the hydrolytic product was diluted two-fold with ultra-pure water and filtered with a 0.2 μm syringe (Millex^®^ hydrophobic PTFE). The HPAEC-PAD system was operated at 30 °C with an isocratic eluent of 150 mM NaOH, at a flow rate of 0.5 mL/min. The standard, including manno-oligosaccharides and galactomanno-oligosaccharides, obtained from Megazyme were used.

### Lab-scale bioconversion of copra mannan into β-MOS

To prepare the substrate, 5 g of DCM containing approximately 2.6 g of mannan were resuspended in 100 mL DI water and incubated at 40 °C with shaking at 250 rpm for 30 min. Then, 5 U/mL of purified β-mannanase (*Bl*Man26B) was added and further incubated for 20 h. To collect the products, the mixture was heated at 100 °C for 20 min, filtered (Whatman No.1 paper), and centrifuged at 2000 × *g* for 10 min. The products were subsequently concentrated, lyophilized, and stored at 4 °C.

### Effects of MOS on bacterial growth

Probiotic bacterial strains including *Bifidobacterium longum* (Omniflora, Fieberbrunn; Austria), *Lactobacillus acidophilus* DSM20079 (German Collection of Microorganisms and Cell Cultures (DSMZ), Braunschweig, Germany), *L. amylovorus* DSM20552 (DSMZ, Braunschweig, Germany), *L. delbrueckii* sub. *bulgaricus* DSM20081 (DSMZ, Braunschweig, Germany), *L. gasseri* (Omniflora, Fieberbrunn, Austria), *Streptococcus salivarius* DSM2059 (DSMZ, Braunschweig, Germany), and *S. thermophilus* APC151 (isolated from Thai organic yogurt company), were grown overnight in MRS basal media under anaerobic conditions. Then, the cultures were centrifugated at 4 °C, 8000 × *g* for 10 min and resuspended in 0.85% NaCl solution to reach an OD_600_ of 0.05. Subsequently, 20 µL of those resuspended cells was added into each well of 96-well microplates containing 180 µL of MRS, and 2% (w/v) oligosaccharides, i.e., fructo-oligosaccharides (FOS, Batch No. SLBQ7714V, Sigma-Aldrich, USA), galacto-oligosaccharides (GOS, Yakult Pharmaceutical, Japan), inulin (Giffarine, Thailand), or manno-oligosaccharides (β-MOS, this study), as carbon sources. The microplates were then anaerobically incubated at 37 °C. A similar experimental setup was also applied for the pathogenic bacterial strains. The pathogenic bacteria are *Escherichia coli* DH5αF (Biolabs; New England) and *Salmonella enterica* subsp. *enterica* serotype *typhimurium* (TISTR No. 2519, TISTR, Thailand), but they were cultured aerobically in M9 minimal broth and incubated at 37 °C, 150 rpm. The inoculated plates were then placed in the reading chamber of a Bioscreen C MBR system (Labsystems, Vantaa, Finland). The optical densities (OD_600_) of the cultures were measured every 30 min for 24 h or 30 h, for probiotic or pathogenic strains, respectively.

### MOS anti-inflammatory assay

The anti-inflammatory activity of β-MOS on THP-1 monocytes was evaluated according to our previous published protocol^22^. The THP-1 cells (4 × 10^5^ cells/well in a 24-well plate) were exposed to 0.2 µM of Vitamin D3 for 48 h (Calbiochem, Germany) to induce differentiation into mature monocytes. After that, the cells were pre-treated with β-MOS at 10, 50, 100, 200, 500, and 1000 µg/mL or with 0.2 µg/mL dexamethasone (Sigma-Aldrich, USA), as a positive control, for 24 h. In the following step, 100 ng/mL of LPS (Invitrogen) was applied to each well and further incubated for 7 h to stimulate inflammatory reactions. Following this step, the culture supernatant from each well was harvested by centrifugation at 700 × g for 5 min and subsequently kept at −20 °C to analyze the amount of secreted IL-1b by ELISA as previously described^22^.

### Cell viability assay

The resazurin analysis was used to investigate the effect of β-MOS on the human dermal fibroblast (HDF) cells following the manufacturer’s instruction (Invitrogen). For each well of 96-well plate, 5 × 10^4^ cells were seeded and incubated at 37 °C (5% CO_2_) for 12–14 h. Then, HDF cells were exposed to β-MOS (62.5, 125, 250, 500, 1000, and 2000 µg/mL) or puromycin (0–8 µg/mL) for 24 h. Following the removal of the media and replacement with 100 µL of 2.5 µg/mL resazurin solution, the plate was subsequently incubated for 3 h to allow a pink color to develop. The absorbance of the reaction was assessed using a microplate reader (BMG Labtech, Germany) at excitation and emission wavelengths of 560 nm and 590 nm, respectively.

The viability of THP-1 cells (Germany) was assessed by MTT assay. An amount of 1 × 10^5^ cells/well of THP-1 monocytes were cultured in RPMI media containing 0.2 µM Vitamin D3 (Merck, UK) at 37 °C for 48 h. After that, 12.5 µL of various concentrations of MOS solutions, ranging from 10, 50, 1000, 200, 500, and 1000 µg/mL were applied to each plate and further incubated for 24 h. Subsequently, RPMI medium was added into each well to reach a total volume of 125 µL per well and incubated for another 7 h. Next, 100 µL of the supernatant was replaced with 100 µL of MTT (Invitrogen, USA) reagent to reach a final concentration of 0.5 mg/mL, and further incubated for another 2 h. The plates were then centrifuged at 700 × *g* for 5 min. Excess MTT solution was discarded, and formazan crystals generated from viable cells were dissolved in 175 µL of solubilizing solution (DMSO). Finally, a microplate reader (Tecan, Austria) was used to measure the absorbance of the reaction at 595 nm. Cell viability was determined by calculating the average absorbance of the samples after subtracting the absorbance of a blank without cells.

### Statistical analysis

The data are presented as mean ± standard deviation (SD) and were evaluated using one-way analysis of variance (ANOVA) and Bonferroni’s multiple comparisons test for parametric data. For non-parametric data, the Kruskal–Wallis test and Dunn’s multiple comparisons test were used, following an indicated normal distribution. Statistical analyses were conducted using GraphPad Prism 8 software (GraphPad Software Inc., California, USA) with the specific tests detailed in the figure legends.

## Results and discussion

### Defatted copra meal preparation and composition

The raw material was collected from a virgin coconut oil factory, of which the copra meal had not been exposed to heat or high pressure. A pre-treatment step involved drying, grinding, and lipid extraction. Two steps were used to remove the remaining fat in the copra meal. The first step involved boiling and refrigeration, of which 80% of the remaining lipid content could be removed. In the second step, *n*-hexane, a food-approved solvent, was used to remove the rest of the oil. The amount of moisture before and after pre-treatment was 48.6 ± 1.2% and 5.7 ± 0.2%, respectively; hence 88% water reduction. The lipid content decreased from 29.4 ± 1.2 to 4.2 ± 0.2%, after the pre-treatment process. Therefore, the total amount of fat removed using one solvent treatment method as described in this study was 86%. The result was less than previous report, of which 97% of fat was removed after three steps of extraction, i.e., boiling and refrigerating, then extraction with methanol or ethanol, followed by ether or *n*-hexane^18^. The de-fatting process has been proposed to be essential for efficient bioconversion of copra meal into β-MOS because the structure of copra meal will be disrupted and more porous, enhancing enzyme accessibility to the mannan polymer^23^. Previously, we showed that without the pre-treatments the β-mannanase from *B. licheniformis*displayed no activity against copra meal^12,13^. Our results, as shown in the next section, indicate that 86% fat removal is sufficient to destabilize the copra meal matrix, enhancing enzyme accessibility, and consequently resulting in an increase in the production of MOS. Table 1 presents the monosaccharide composition of DCM. Mannose is the major sugar, comprising 52.08% (w/w of dry weight), followed by other monosaccharides. These data suggest that defatted copra meal, with its elevated mannan content, can be a preferred substrate for MOS production using enzyme technology. For the economical point of view, although the heat treatment and solvent extraction process will require some expenses, the solvent can be reused. Most importantly, it is expected that the value added to the final product can offset the costs incurred during production due to the low cost of raw materials and the high applicability of the product at a very small quantity.

**Table 1:** The monosaccharides profile in defatted copra meal.

Monosaccharide	% (w/w)*
Mannose	52.08
Glucose	5.05
Xylose	1.92
Arabinose	0.60
Galactose	N/A**

*Dry weight.

**N/A: Not available because the Galactose and Mannose peaks are overlapped in the Aminex HPX42A column which is used in this study.

### Enzymatic production of β-MOS

**Fig. 1:**
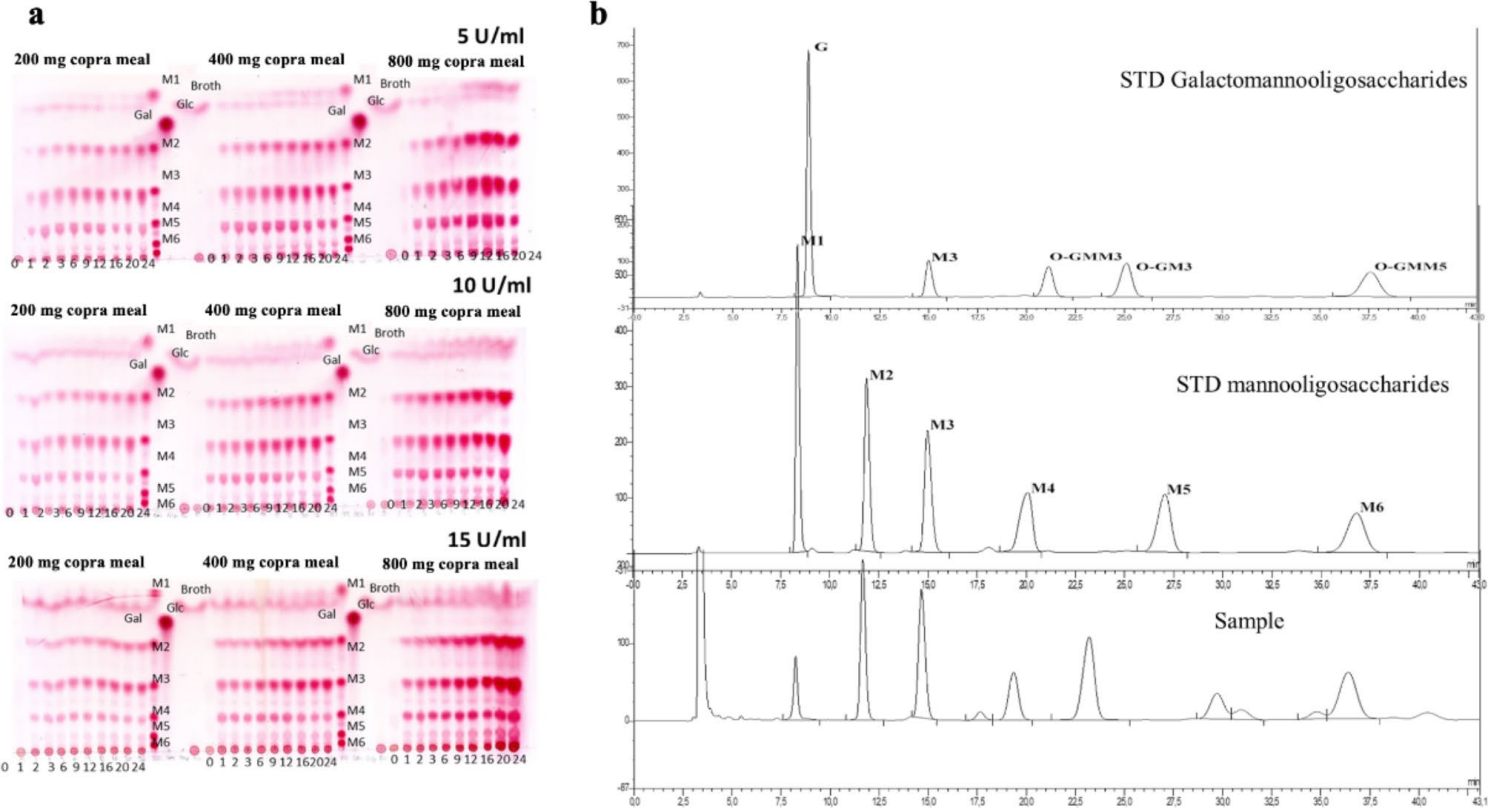
Optimization of bioconversion reaction and analysis of β-MOS products. (**a**) TLC analysis of products from various reaction conditions of substrate and enzyme at various time points (0, 1, 2, 3, 6, 9, 12, 16, 20, and 24 h). (**b**) HPAEC-PAD analysis of MOS composition at optimal reaction conditions. Galactomanno-oligosaccharides and manno-oligosaccharides standards were used for comparison and product quantitation. The corresponding retention times observed were 11.67 min for mannobiose, 14.65 min for mannotriose, 19.35 min for mannotretraose, and 36.42 min for mannohexaose. O-GMM3, 6^1^-α-D-Galactosyl-mannotriose; O-GM3, 6^1^-α-D-Galactosyl-mannobiose plus mannotriose.

Different reaction conditions, employing various substrate concentrations ranging from 200, 400, and 800 mg/mL (corresponding to 1.0, 2.1 and 4.2% of total mannan) and enzyme doses (5, 10, and 15 U/mL of reaction mixture) were investigated to obtain the optimal bioconversion conditions in terms of product yield and product spectrum. Even if the optimal temperature of *Bl*Man26B was at 50 °C, these reactions were carried out at 40 °C, of which ~ 75% relative activity was previously observed^14,24^. Reducing the reaction temperature 10 ℃ will be more economical for large-scale bioconversion reactions due to lower energy consumption. This temperature is also close to ambient temperature in many tropical countries all year round. The hydrolysis of copra meal catalyzed by purified *Bl*Man26B as analyzed by TLC and HPAEC-PAD is shown in Fig. 1a and b, respectively. These results indicate that *Bl*Man26B was able to break down copra meal mannan into manno-oligosaccharides (MOS) comprising mannobiose (M2), mannotriose (M3), mannotetraose (M4), mannopentaose (M5) and mannohexaose (M6), as previously observed^12^. Notably, the position of M5 did not correspond well with the standard. This product could be either α-1,6-galactosylated or glucosylated derivative of a manno-oligosaccharide. The hydrolysis product also showed several small peaks of DP 3–6 unidentified sugars and a trace of mannose (M1). This result is different from that obtained when using the enzyme from *Clostridium thermocellum* (*Ct*ManT)^25^, of which M1-M3 were the main products. At the optimal condition, the yield of copra meal mannan conversion was 36.7%, which was obtained after 16 h of reaction with an initial copra meal concentration of 400 mg/mL (corresponding to 2.1% of total mannan) and 5 U of *Bl*Man26B per mL of reaction mixture. This result is significantly higher than 8.3% yield reported for MOS bioconversion from copra meal (without defatting stage) using commercial enzyme Pectinex^®^ Ultra SP-L, which is a blend of β-mannanase, pectinases, hemicellulases and beta-glucanases from *Aspergillus aculeatus*^26^, but slightly lower than 38.99% of total MOS yield from defatted copra meal using enzyme cocktail (β-mannanase, cellulase, xylanase, paperase, β-glucosidase, β-xylosidase and α-galactosidase) from *A. quadrilineatus* RSNK-1^27^. MOS yield may increase with increasing initial copra meal concentration. However, it may decrease at too high copra meal concentration due to the diffusion limitation of the enzyme in the high viscosity of substrate, which may cause inhibition to the enzyme activity. This is consistent with the conclusion that increasing galactomannan concentration (> 5.0%, w/v) led to decreasing the yield of hydrolysis products^28^. Subsequently, lab-scale production of β-MOS for further analysis was conducted using 5% of copra meal and 5 U/mL of enzyme at 40 °C for 20 h, as this appeared to be the optimal bioconversion condition. After the enzymatic conversion process, the β-MOS was recovered simply by filtration (Whatman No.1), followed by 10 min centrifugation at 2000 × *g*, and lyophilization. The products were kept at room temperature in a desiccator for functional analysis in the following sections.

### Fermentability of β-MOS

**Fig. 2:**
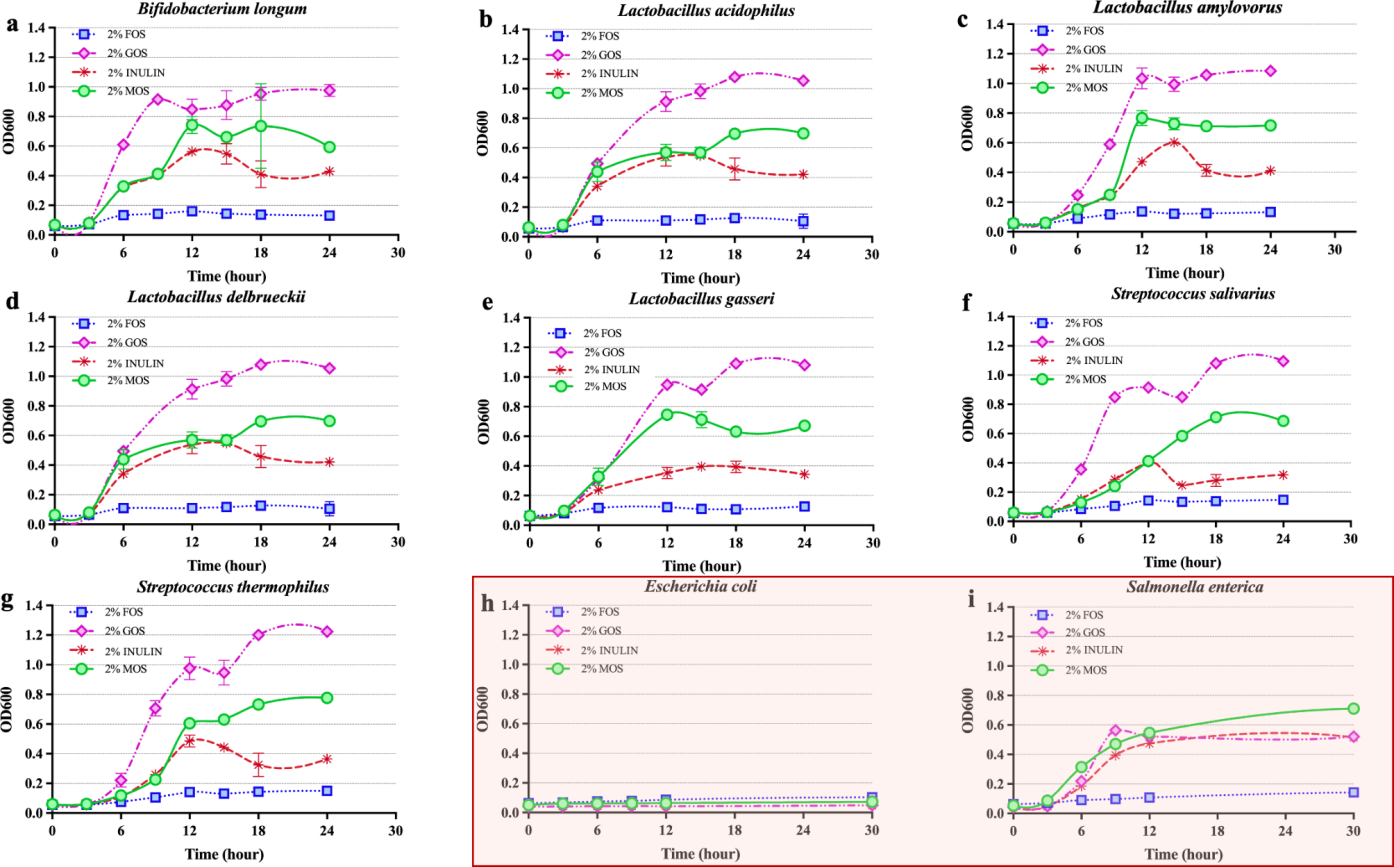
Promotion of growth of probiotics and potentially pathogenic bacteria by β-MOS and other oligosaccharides. Growth of the probiotic bacteria *B. longum* (**a**), *L. acidophilus* (**b**), *L. amylovorus* (**c**), *L. delbrueckii* (**d**), *L. gasseri* (**e**), *S. salivarius* (**f**) and *S. thermophilus* (**g**) and pathogenic bacteria *E. coli* (**h**) *and S. enterica* (**i**) was monitored when cultured in media containing fructooligosaccharides (FOS, squares), galactooligosaccharides (GOS, diamonds), inulin (asterisks) and β-MOS (circles). The data are given as mean ± SD (*n* = 3).

Next, specific growth stimulation of certain probiotic strains by our β-MOS mixture was investigated as illustrated in Fig. 2a-g. The time course of the single-strain fermentations of *B. longum*, *L. acidophilus* DSM20079, *L. amylovorus* DSM20552, *L. delbrueckii* sub. *bulgaricus* DSM20081, *L. gasseri*,* S. salivarius* DSM2059 and *S. thermophilus* APC151 on various oligosaccharides including β-MOS (2% w/v) were compared. The highest growth of all tested strains was observed when GOS (containing ~ 20% glucose) was used as the C-source, followed by MOS, inulin, and FOS. These results indicate the potential of β-MOS to promote growth of certain probiotic bacterial strains. The growth of *S. enterica* serotype *typhimurium* on MOS, inulin and GOS was comparable to that of the probiotic tested strains, only in the presence of FOS the growth of this pathogenic strain seems lower than the above-mentioned tested strains (Fig. 2i). None of the oligosaccharides could support the growth of *E. coli* in aerobic conditions (Fig. 2h). These results confirmed several previous reports on the prebiotic potential of β-MOS generated by bioconversion of copra meal using different endo β-mannanases. These included enzymes from various sources such as *B. circulans* NT 6.7^29^, *Clostridium thermocellum* ATCC 27,405^25^, *Aspergillus niger*^30^, *Bacillus* sp. SWU60 (rManS2)^31^, *B. subtilis* MAN7^32^, and *Klebsiella oxytoca* KUB-CW2-3^33^. These reports, in addition to showing enhanced growth of potentially probiotic strains, indicated other important characteristics of MOS such as resistance to degradation by the digestive system^25^, enhancing epithelial tight junction integration^34^, and other beneficial health benefitsfor humans and animals^35,36^. However, so far there have been no reports on cytotoxicity nor anti-inflammatory activity of β-MOS at the cellular level, which will be revealed in the following sections of this study.

### The effect of β-MOS on the viability of human skin (HDF) and monocytes

**Fig. 3:**
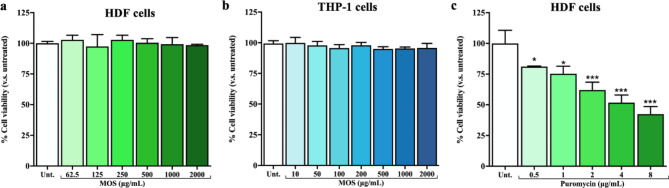
Viability of HDF and THP-1 cells in the presence of β-MOS. β-MOS were used at 62.5–2000 µg/mL and incubated for 24 h on HDF cells (**a**) and at 10–2000 µg/mL for THP-1 cells (**b**). Puromycin (**c**) was used at 0.5–8 µg/mL as a control. Cell viability was tested by the resazurin assay and the MTT assay for HDF cells and THP-1 cells, respectively. The absorbance of the untreated cells was used as a reference, representing 100% viability. Each bar represents the average value of triplicates and the error bar represents the standard deviation of the mean. Statistical analysis versus untreated was performed using One-way ANOVA, Bonferroni’s multiple comparisons test: *P < 0.05, **P < 0.01, ***P < 0.001.

The effects of β-MOS at various concentration ranging from 0 to 2000 µg/mL on the viability of HDF and mature human monocytes (THP-1), which are the representative of human skin and immune system, are reported in Fig. 3a and b, respectively. As illustrated, no cytotoxicity was observed even at the highest concentration of β-MOS employed on both the HDF and THP-1 cells. When HDF cells were treated with 0.5–8 µg/mL of puromycin, a cytotoxic drug known to inhibit protein synthesis, gradual reduction of viability was observed (Fig. 3c). These results confirm previous reviews indicating that β-MOS are safe to be used as feed additives, cosmeceuticals or nutraceuticals^9,27^. Notably, the cytotoxic test on human dermal fibroblast is preferable for cosmetic products instead of animal testing^37^.

### Anti-inflammatory assay of MOS on human monocytes

**Fig. 4:**
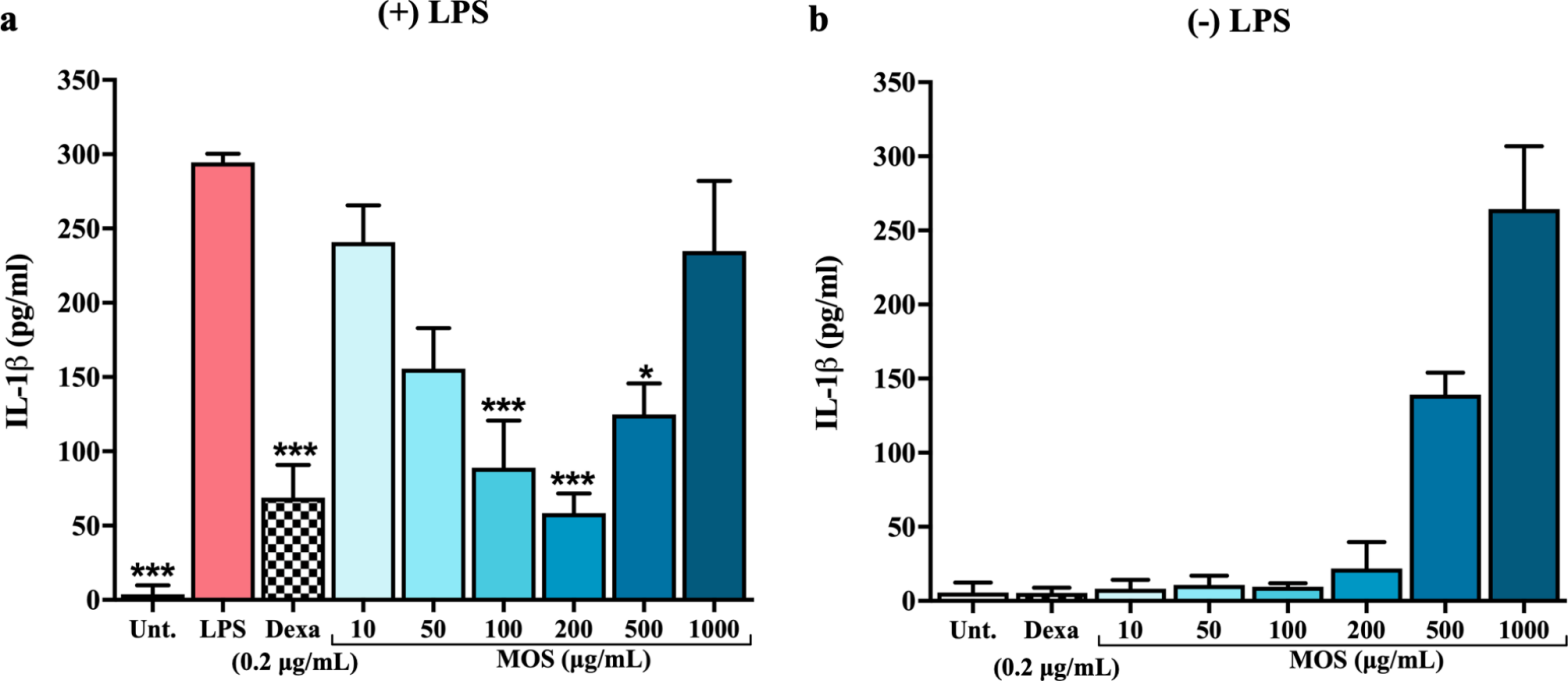
Anti-inflammatory effects of β-MOS. The anti-inflammation was measured using VitD3-differentiated THP-1 cells. The cells were pretreated with β-MOS at concentrations ranging from 10 to 1000 µg/mL, while 0.2 µg/mL dexamethasone was used as a control. LPS was used to induce the inflammation of differentiated THP-1 cells. Each bar represents the average value of quadruplicates and the error bar represents the standard deviation of the mean. Statistical analysis was performed using One-way ANOVA, Bonferroni’s multiple comparisons test: *P < 0.05, **P < 0.01, ***P < 0.001. .

The anti-inflammatory effects of β-MOS were observed on mature human monocytes. THP-1 cells were first induced to differentiate by Vit D3 to simulate primary human monocytes^22^. Then, the THP-1 cells were pre-treated with β-MOS (0 to 1,000 µg/mL) for 24 h. After that, the cells were challenged with bacterial lipopolysaccharides (LPS) for 7 h. Controls are the cells treated with steroid dexamethasone. The amount of pro-inflammatory cytokine IL-1β that was released into culture media was analyzed by ELISA as shown in Fig. 4a. While LPS could significantly increase IL-1β secretion from differentiated THP-1 (*p* < 0.05), the cells that were pre-treated with β-MOS showed a significant does-dependent reduction in IL-1β secretion. The anti-inflammatory effects of β-MOS were noticeable even at concentrations as low as 10 µg/mL, with effectiveness gradually increasing as the dosage of β-MOS was raised, reaching its peak at 200 µg/mL. MOS at 100–200 µg/mL (0.01–0.02%) could significantly (*p* < 0.001) inhibit IL-1β release. The maximum impact reached with these β-MOS is akin to the effect of dexamethasone at a concentration of 0.2 µg/mL. On the other hand, β-MOS at 500-1,000 µg/mL appeared to stimulate the release of IL-1β in cells not exposed to LPS (Fig. 4b). These findings suggest that the optimal dosage of β-MOS needs to be meticulously assessed to achieve maximal benefit, as higher doses of β-MOS may exacerbate inflammation. Remarkably, the U-shaped dose-response curve found in this study is a popular phenomenon in various toxicological, biological, and pharmacological studies, including the effect of chito-oligosaccharides (COS)^22^. This represents the first report on the potential of β-MOS in exerting anti-inflammatory effects at the cellular level. The high potency of β-MOS as novel anti-inflammatory natural product at 0.01–0.02% ensure the cost-effectiveness of the valorization process as described in this study.

### A bioprocess to produce functional MOS using enzyme technology

**Fig. 5:**
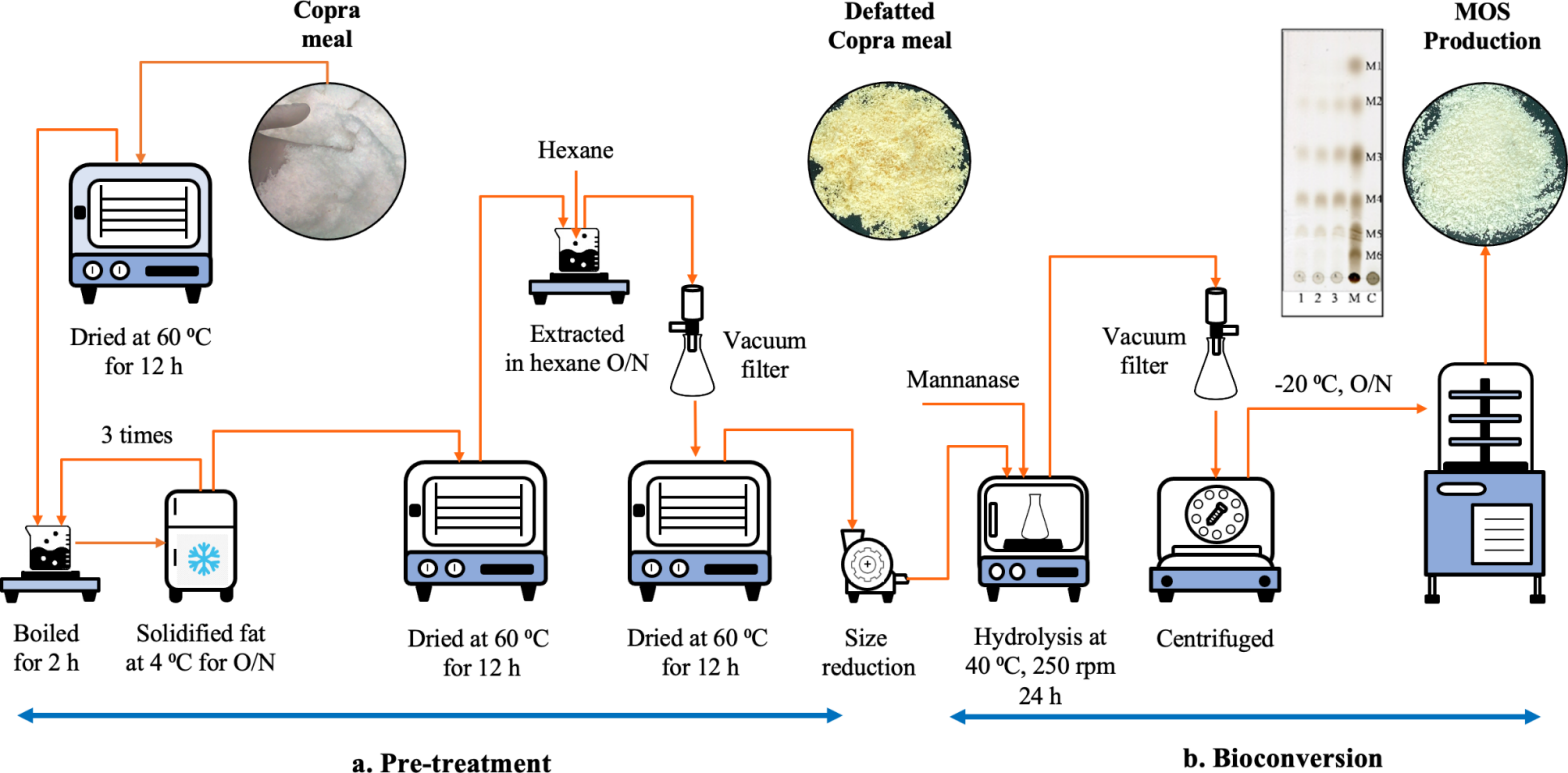
Schematic diagram for the production of β-MOS using enzyme technology. (**A**) Pretreatment. The copra meal sample was heated in a hot air oven at 60 °﻿C for 12 h. For the defatting process, copra meal was boiled for 2 h in twice the volume of DI water, and then kept in 4 °﻿C for solidification and removal of the remaining coconut oil. This process was repeated three times. After that the sample was dried again in a hot air oven for 12 h at 60 °﻿C, before suspending in 1 L of *n*-hexane with stirring overnight. Then the *n*-hexane was removed by vacuum filtration and the defatted copra meal (DCM) was dried at 60 °C for 12 h. Then the DCM was ground, sieved through 0.2 mm mesh, and kept in 4 °﻿C until used. (**B**) Bioconversion. Copra meal was suspended in DI water and β-mannanase was added to the reaction at 500 U per 2.6 g mannan. The reaction mixture was incubated for 24 h at 40 °﻿C, 250 rpm. The soluble β-MOS product in the supernatant was collected after filtration, centrifugation, and kept at −20 °﻿C overnight before lyophilization. Pictures of copra meal waste from the virgin coconut oil company, de-fatted copra meal after pre-treatment, and lyophilized MOS from this study are shown.

A schematic diagram summarizing the process of β-MOS production from copra meal, which was designed to be suitable for functional food, cosmeceuticals, or nutraceutical products, is shown in Fig. 5. The copra meal waste from the virgin coconut oil factory appeared clean. It is normally sold as cheap, low-nutrition animal feed or ended up in landfill. The process involved two main parts, i.e., (a) pre-treatment and (b) bioconversion. The pre-treatment step deals with a decrease of moisture content, lipid extraction, and size reduction, thereby promoting the enzymatic action. The bioconversion step involves enzymatic hydrolysis of mannans from copra meal into β-MOS, using the enzyme *Bl*Man26B, followed by the recovery of the β-MOS products, mainly composed of M2 to M6, by filtration, centrifugation, and lyophilization. Notably, no further purification step was applied. The enzyme *Bl*Man26B, which is suitable for industrial application in this study, was originally obtained from *B. licheniformis* and recombinantly produced from *E. coli*^12^ or *L. plantarum*, the food-grade expression systems^14^. The enzyme from both expression systems can be used to produce β-MOS without any significant difference in either its physicochemical or biological properties.

While the valorization process as described in this study can’t be regarded as “green” technology because hexane was used to extract the residue oil, in the future an alternative green solvent from nature (water, CO_2_) or terpenes (*d*-limonene, *p*-cymene and α-pinene) from agricultural residues can be used to replace hexane^38^, allowing the process to be more environmental friendly.

## Conclusions

**Fig. 6:**
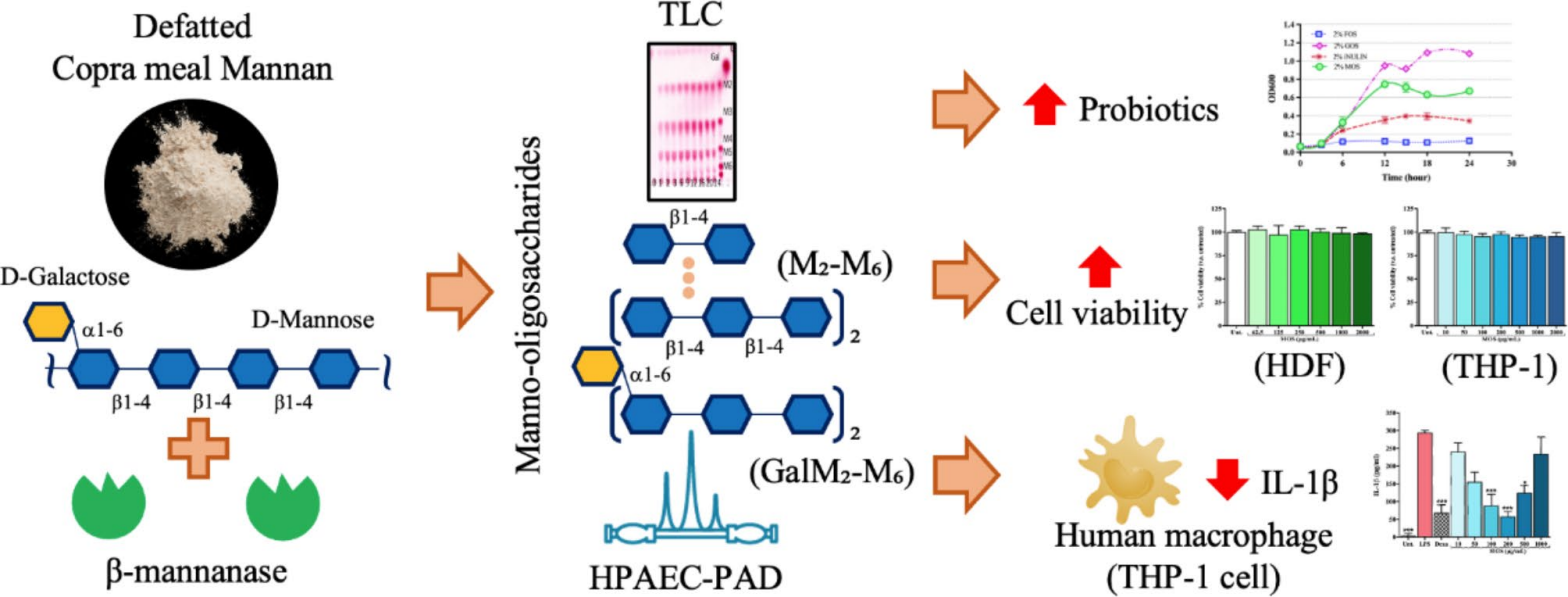
A summary of this research. The bioconversion of defatted copra meal from virgin coconut oil manufacturing into functional manno-oligosaccharides (β-MOS) by enzymatic hydrolysis using β-mannanase from *Bacillus licheniformis* (*Bl*ManB) was investigated in this study. The size of oligosaccharides, analyzed by TLC and HPLC, obtained ranged from dimers to hexamers with 36.7% conversion yields. The in vitro growth promoting effects of β-MOS were demonstrated on various probiotic bacteria compared to other oligosaccharides. In vitro toxicity assays of β-MOS on human dermal fibroblasts and monocytes showed no cytotoxic effect. For the first time, β-MOS at a concentration ranging from 10 to 200 µg/mL also demonstrated potent anti-inflammatory activity against LPS-induced inflammation of human macrophage THP-1, in a dose-dependent manner.

In summary (Fig. 6), a bioconversion process to produce bioactive manno-oligosaccharide from defatted copra using *Bl*Man26B from *B. licheniformis* was established. In addition to a proven prebiotic effect, the β-MOS in this study also showed additional attractive properties as indicated by their potent dose-dependent anti-inflammatory activity in LPS-induced THP-1 monocytes. Moreover, while no toxicity in both human skin cells and monocytes was observed at high doses, β-MOS could also stimulate inflammation at these high concentrations. Therefore, further investigation must be conducted to ensure its efficacy and safety uses in the future. In addition, the process needs to be optimized so that the production cost will be effective for a large-scale bioconversion of copra meal wastes into functional β-MOS in the next step. These findings should be applicable to other agricultural wastes rich in mannan and galactomannan or glucomannan such as coffee bean, palm kernel, konjac, or locust bean gum as well.

## Data Availability

Correspondence and requests for materials should be addressed to the corresponding authors: Montarop Yamabhai and Thu-Ha Nguyen.
