# Author Correction: Value creation of copra meal mannan into functional manno-oligosaccharides (β-MOS) using the mannanase *Bacillus* man B (*Bl*Man26B)

**DOI:** 10.1038/s41598-024-76755-6

**Published:** 2024-11-04

**Authors:** Nguyen Cao Cuong, Dietmar Haltrich, Thae Thae Min, Thu-Ha Nguyen, Montarop Yamabhai

**Affiliations:** 1 Faculty of Engineering and Food Technology, Hue University of Agriculture and Forestry, Hue University, Thua Thien Hue, Hue, 530000 Vietnam; 2 Molecular Biotechnology Laboratory, School of Biotechnology, Institute of Agricultural Technology, Suranaree University of Technology, Nakhon Ratchasima, 30000 Thailand; 3 Food Biotechnology Laboratory, Department of Food Science and Technology, BOKU University, 1190 Vienna, Austria

Correction to: *Scientific Reports* 10.1038/s41598-024-73255-5, published online 27 September 2024

The Acknowledgments section in the original version of this Article was incomplete.

“A part of this research was funded by the Royal Golden Jubilee scholarship (RGJ-Ph.D/0108/2552) for Mr. Suttipong Sak-Ubol’s Ph.D. thesis. Other financial supports include Forthcoming Research to Industry Convergence (grant number FSUT3-304-63-12-3), Thailand Science Research and Innovation (TSRI), National Science, Research and Innovation Fund (NRSF), Grant no. 179306, 4693955, FF3-304-66-12-200(H31), FF6-614-66-24-38(H), the Austrian Science Fund (FWF Projects P 37092-B and P 35611-B), and the postdoc grant no. Full-time 66/12/2024 supported by Suranaree University of Technology, Thailand. The authors acknowledge Thai Pure Coconut Company Limited for providing the copra meal. The authors would also like to thank Dr. Munthipha Khamphio, and Mr. Phurich Ariyarak for excellent technical assistances.”

now reads:

“A part of this research was funded by the Royal Golden Jubilee scholarship (RGJ-Ph.D/0108/2552) for Mr. Suttipong Sak-Ubol’s Ph.D. thesis. Other financial supports include Forthcoming Research to Industry Convergence (grant number FSUT3-304-63-12-3), Thailand Science Research and Innovation (TSRI), National Science, Research and Innovation Fund (NRSF), Grant no. 179306, 4693955, FF3-304-66-12-200(H31), FF6-614-66-24-38(H), and the postdoc grant no. Full-time 66/12/2024 supported by Suranaree University of Technology, Thailand. The authors acknowledge Thai Pure Coconut Company Limited for providing the copra meal. The authors would also like to thank Dr. Munthipha Khamphio, and Mr. Phurich Ariyarak for excellent technical assistances. This research was funded in whole or in part by the Austrian Science Fund (FWF) [grant DOI: 10.55776/P35611]. For open access purposes, the author has applied a CC BY public copyright license to any author accepted manuscript version arising from this submission."

Additionally, this Article was incorrectly published under CC BY-NC-ND licence. The licence has been corrected to CC BY.

The original Article has been corrected.

